# Esophageal myotomy and risk of esophageal cancer and mortality in achalasia: Real-world cohort study

**DOI:** 10.1055/a-2801-4957

**Published:** 2026-03-16

**Authors:** Fouad Jaber, Mohamad Ali Ibrahim, Mohammed Jaber, Youssef Soliman, Mai A Khalaf, Tara Keihanian, Fares Ayoub, Mohamed O. Othman, Salmaan Jawaid

**Affiliations:** 1 3989 Division of Gastroenterology and Hepatology, Baylor College of Medicine, Houston, United States; 2 3989 Department of Medicine, Baylor College of Medicine, Houston, United States; 3 123683 Faculty of Medicine, Al-Azhar University, Gaza, Palestine; 4 68796 Assiut University, Asyut, Egypt

**Keywords:** Endoscopy Upper GI Tract, Motility / achalasia, Barrett's and adenocarcinoma, POEM

## Abstract

**Background and study aims:**

Achalasia is associated with increased risk of esophageal cancer, particularly squamous cell carcinoma. Although esophageal myotomy improves dysphagia, its impact on cancer risk and mortality remains unclear.

**Patients and methods:**

We conducted a retrospective cohort study using the TriNetX research network, including adults (≥ 18 years) with achalasia confirmed by esophageal manometry. Patients were categorized based on treatment of those undergoing esophageal myotomy (peroral endoscopic myotomy [POEM] or laparoscopic Heller myotomy [LHM]) and those managed without myotomy. Patients with prior esophagectomy or malignancies associated with increased esophageal cancer risk were excluded. The primary outcome was incident esophageal cancer; secondary outcomes included all-cause mortality. Propensity score matching balanced baseline characteristics. Associations were assessed using adjusted odds ratios (aORs) and Cox proportional hazards models. Overall survival was assessed using Kaplan–Meier analysis and compared with the log-rank test.

**Results:**

Among 18,186 patients with achalasia, 3,758 underwent esophageal myotomy and 14,428 were managed without myotomy. After matching, esophageal cancer incidence was low and did not differ significantly between the myotomy and non-myotomy cohorts (0.29% vs 0.27%; aOR 1.1, 95% confidence interval [CI] 0.47–2.6). In contrast, myotomy was associated with significantly lower all-cause mortality (3.25% vs 7.22%; aOR 0.43, 95% CI 0.35–0.54). Independent predictors of esophageal cancer included male sex, older age, and personal history of gastrointestinal malignancy.

**Conclusions:**

In short-term follow-up, esophageal myotomy in achalasia was associated with lower all-cause mortality and similar esophageal cancer incidence. These findings suggest benefits beyond symptom control, including a potential survival advantage.

## Introduction

Achalasia is an esophageal motility disorder characterized by insufficient relaxation of the lower esophageal sphincter (LES) and impaired esophageal peristalsis. Although rare, achalasia is a debilitating condition that primarily affects a relatively young patient population, with evidence suggesting rising incidence and prevalence 1 . Clinical manifestations include dysphagia for solids and liquids, regurgitation, chest pain, heartburn, and respiratory symptoms 2 . Pathophysiology involves inflammation and degeneration of the inhibitory neurons in the myenteric plexus, although the underlying etiology of this process remains unknown 3 .

Because no curative treatment is available yet, management aims to relieve symptoms by decreasing resting pressure of the LES to facilitate passage of ingested food. Treatment strategies range from pharmacological reduction of LES pressure with nitrates, calcium-channel blockers, or botulinum toxin injection, to mechanical disruption of LES muscle fibers with endoscopic pneumatic dilation, surgical laparoscopic Heller myotomy (LHM) with a partial fundoplication, or peroral endoscopic myotomy (POEM) 4 . LHM was considered the gold standard treatment for achalasia for many years. Recently, however, POEM has recently emerged as a safe and effective treatment for achalasia, demonstrating similar short and middle-term symptomatic control to LHM 5 6 . Achalasia-induced stasis can lead to bacterial overgrowth, chemical irritation, and chronic esophageal inflammation, which may promote esophageal squamous cell carcinoma (ESCC) 7 8 9 . ESCC typically develops a decade after diagnosis, although earlier onset is possible 10 . The prognosis of ESCC in achalasia patients is considered poor because they are used to a certain degree of dysphagia, which tends to delay diagnosis until advanced stages 11 . Conversely, myotomy—by lowering LES pressure—can induce gastroesophageal reflux, increasing risk of Barrett’s esophagus (BE) and esophageal adenocarcinoma (EAC), particularly after POEM, which lacks fundoplication 5 6 12 13 . Nevertheless, by relieving chronic stasis, myotomy may theoretically reduce mucosal exposure to retained food and fluid, potentially lowering ESCC risk.

Although the link between achalasia and esophageal cancer (EC) has been reported in case series and cohort studies 14 15 16 17 , risk of developing it following esophageal myotomy remains poorly understood. This study aimed to assess whether esophageal myotomy (LHM or POEM) influences risk of EC and all-cause mortality in patients with achalasia.

## Patients and methods

### Data source

This retrospective cohort study utilized data from the TriNetX US platform, a federated network that aggregates deidentified electronic health records from over 60 healthcare systems across all 50 states. The database draws from a diverse range of institutions, including academic hospitals, integrated healthcare systems, and outpatient practices. It provides structured, standardized information on demographics, diagnoses (ICD-10-CM), procedures (Current Procedural Terminology [CPT], ICD-10-PCS), medications (RxNorm, Veterans Affairs Drug Classification), laboratory findings (LOINC), vital signs, clinical visits, and healthcare use, including hospital admissions. Data undergo rigorous quality checks to ensure accuracy and completeness before being incorporated into the platform. All records are deidentified following Health Insurance Portability and Accountability Act standards and only aggregate-level data are available to researchers. Consequently, research using TriNetX data is generally classified as non-human subjects research and is exempt from institutional review board approval.

### Study population

A real-time search and analysis were performed covering the period from January 1, 2004, to December 31, 2024. Adult patients (aged ≥ 18 years) with a diagnosis of achalasia were identified for inclusion. To improve diagnostic accuracy, inclusion was restricted to patients who underwent confirmatory esophageal manometry, identified using relevant CPT codes for esophageal manometry (0240T, 91010).

Patients were categorized into two groups based on treatment strategy: 1) those who underwent esophageal myotomy, including LHM or peroral endoscopic myotomy (POEM); and 2) those managed without myotomy (non-myotomy group), which included patients treated with alternative achalasia therapies such as pneumatic dilation or botulinum toxin injection.

Patients with a prior esophagectomy were excluded. To reduce misclassification from secondary achalasia due to malignancy, we required a minimum 6-month lead time following achalasia diagnosis and excluded patients with a history of malignancies associated with increased EC risk, including head and neck, lung, breast, and gastric cancers. All exclusion criteria were applied prior to the index date to ensure appropriate temporal sequencing and minimize selection bias. ICD-10-CM and CPT codes used to define diagnoses and procedures are provided in Supplementary Table 1.

### Study outcomes

The primary outcome was incidence of EC among patients with achalasia. Secondary outcomes included all-cause mortality, incidence of gastroesophageal reflux disease (GERD), and BE. Outcomes were identified using validated ICD-10-CM codes (Supplementary Table 1)

### Statistical analysis

All statistical analyses were conducted using the browser-based analytics tools within the TriNetX Live platform (TriNetX LLC, Cambridge, Massachusetts, United States).

### Propensity score matching

Propensity score matching (PSM) was performed to minimize baseline differences between the myotomy and non-myotomy groups. Propensity scores were generated using logistic regression models and included demographics (age, sex, race, ethnicity, and body mass index [BMI]), behavioral risk factors (tobacco use, alcohol use), comorbidities (GERD, hepatic cirrhosis, chronic kidney disease [CKD], chronic obstructive pulmonary disease [COPD], heart failure, type 2 diabetes mellitus [T2DM], hypertensive diseases, and hyperlipidemia), medication exposures (proton pump inhibitors [PPIs] and opioid use disorder), and baseline endoscopic surveillance frequency, measured by the number of prior esophagogastroduodenoscopies (EGDs). A 1:1 nearest-neighbor matching algorithm with a caliper of 0.1 pooled standard deviations was applied, consistent with TriNetX default PSM parameters.

### Index date and immortal time bias mitigation

The index date for all patients was defined as the date of achalasia diagnosis. Patients were required to have at least 365 days of look-back prior to the index date for baseline covariate assessment. To minimize immortal time bias and ensure a comparable time zero between treatment groups, a 6-month landmark design was used. Follow-up for outcome ascertainment began at the 6-month landmark and continued until outcome occurrence, death, last clinical encounter, or the end of the study period.

### Comparative analyses

The primary analysis compared patients who underwent esophageal myotomy with those who did not undergo myotomy, including medical management, endoscopic therapies, or observation. A secondary analysis compared patients undergoing myotomy with those receiving active non-myotomy interventions, including botulinum toxin injection, endoscopic dilation, or pneumatic dilation. Subgroup analyses were performed to compare myotomy type (POEM vs LHM) with the non-myotomy group, followed by a direct comparison between POEM and LHM.

Associations between myotomy and clinical outcomes were evaluated using Cox proportional hazards models to estimate adjusted hazard ratios (aHRs) with 95% confidence intervals (CIs). For outcomes not suitable for time-to-event analysis, adjusted odds ratios (aORs) were calculated. All models were adjusted for demographic variables, comorbidities, and medication exposures included in the matching procedure. Two-sided *P* < 0.05 was considered statistically significant. Differences in follow-up duration between groups were addressed using time-to-event analyses, including Cox proportional hazards models, which account for varying lengths of follow-up. Follow-up time was calculated from the index date until the earliest of outcome occurrence, last clinical encounter, or end of the study period. Overall survival was estimated using Kaplan-Meier methods, with comparisons between groups performed using the log-rank test.

## Results

### Baseline characteristics

A total of 3,758 patients with achalasia underwent esophageal myotomy, whereas 14,428 were managed without myotomy. Among patients undergoing myotomy, 1,159 underwent POEM and 2,634 underwent LHM. Within the non-myotomy cohort, 2,257 patients received active non-myotomy interventions, including dilation or botulinum toxin injection, whereas the remaining patients were managed with non-procedural approaches, including medical therapy and/or observation.

Baseline characteristics were well balanced between myotomy and non-myotomy cohort: mean age was similar between the myotomy and non-myotomy groups (53.6 ± 16.5 vs. 53.6 ± 18.6 years; *P* = 0.98), as was sex distribution (47.8% vs. 48.7% female; *P* = 0.45). PPI use did not differ significantly between the groups (54.1% vs. 55.3 %; *P* = 0.31) ( Table 1 ). Other demographic variables and comorbidities were also comparable between cohorts.

**Table:** **Table 1** Baseline characteristics of included cohorts before and after matching.

	**Before matching**			**After matching**		
**Variable**	**Myotomy cohort**	**Non-myotomy cohort**	***P* value **	**Myotomy cohort**	**Non-myotomy cohort**	***P* value **
**Number of patients**	3,758	14,438	—	3,754	3,754	—
**Demographics**						
**Age at Index (mean ± SD)**	53.5 ± 16.5	58.8 ± 17.6	< 0.0001	53.6 ± 16.5	53.6 ± 18.6	0.976
**BMI (mean ± SD)**	27.8 ± 6.7	28.3 ± 7.3	0.0002	27.8 ± 6.7	28.2 ± 7.4	0.015
**Gender**						
Male	1,961 (52.2%)	6,294 (43.6%)	< 0.0001	1,957 (52.1%)	1,924 (51.3%)	0.446
Female	1,796 (47.8%)	8,133 (56.3%)	< 0.0001	1,796 (47.8%)	1,829 (48.7%)	0.446
Unknown gender	10 (0.3%)	11 (0.1%)	0.002	10 (0.3%)	10 (0.3%)	1.000
**Race**						
White	2,677 (71.2%)	9,720 (67.3%)	< 0.0001	2,674 (71.2%)	2,743 (73.1%)	0.076
Black or African American	519 (13.8%)	1,964 (13.6%)	0.741	519 (13.8%)	515 (13.7%)	0.893
Asian	89 (2.4%)	389 (2.7%)	0.266	89 (2.4%)	86 (2.3%)	0.819
Unknown race	286 (7.6%)	1,801 (12.5%)	< 0.0001	286 (7.6%)	245 (6.5%)	0.065
Other race	159 (4.2%)	472 (3.3%)	0.004	158 (4.2%)	140 (3.7%)	0.287
**Ethnicity**						
Hispanic or Latino	298 (7.9%)	946 (6.6%)	0.003	297 (7.9%)	273 (7.3%)	0.296
Not Hispanic or Latino	2,797 (74.4%)	10,906 (75.5%)	0.160	2,795 (74.5%)	2,818 (75.1%)	0.541
American Indian or Alaska Native	18 (0.5%)	49 (0.3%)	0.208	18 (0.5%)	14 (0.4%)	0.479
Native Hawaiian or other Pacific Islander	10 (0.3%)	43 (0.3%)	0.748	10 (0.3%)	11 (0.3%)	0.827
Unknown ethnicity	663 (17.6%)	2,586 (17.9%)	0.702	662 (17.6%)	663 (17.7%)	0.976
**Behavioral risk factors**						
Tobacco use	89 (2.4%)	204 (1.4%)	< 0.0001	88 (2.3%)	75 (2.0%)	0.303
Alcohol use	28 (0.7%)	54 (0.4%)	0.003	27 (0.7%)	25 (0.7%)	0.781
**Comorbidities**						
GERD	1,983 (52.8%)	6,638 (46.0%)	<0.0001	1,982 (52.8%)	2,041 (54.4%)	0.172
Hypertensive diseases	1,269 (33.8%)	4,480 (31.0%)	0.001	1,267 (33.8%)	1,261 (33.6%)	0.884
Hyperlipidemia	692 (18.4%)	2,466 (17.1%)	0.054	690 (18.4%)	653 (17.4%)	0.265
Type 2 diabetes mellitus	460 (12.2%)	1,747 (12.1%)	0.814	460 (12.3%)	446 (11.9%)	0.620
Chronic kidney disease	211 (5.6%)	947 (6.6%)	0.035	211 (5.6%)	219 (5.8%)	0.691
COPD	199 (5.3%)	1,024 (7.1%)	< 0.0001	199 (5.3%)	199 (5.3%)	1.000
Heart failure	154 (4.1%)	807 (5.6%)	0.0003	154 (4.1%)	153 (4.1%)	0.954
**Medications**						
Opioid-related disorders	48 (1.3%)	201 (1.4%)	0.589	48 (1.3%)	57 (1.5%)	0.376
Proton pump inhibitor use	2,034 (54.1%)	6,514 (45.1%)	< 0.0001	2,031 (54.1%)	2,075 (55.3%)	0.308
**Others**						
Esophagogastroduodenoscopy	2,314 (61.6%)	5,087 (35.2%)	< 0.0001	2,310 (61.5%)	2,244 (59.8%)	0.119
BMI, body mass index; COPD, chronic obstructive pulmonary disease; GERD, gastroesophageal reflux disease; SD, standard deviation.						

Before PSM, patients in the POEM/LHM cohort had a mean follow-up of approximately 3.38 years and a median follow-up of 2.08 years. In contrast, patients in the non-myotomy cohort demonstrated longer follow-up, with a mean of approximately 3.90 years and a median of 3.08 years. After PSM, follow-up duration in the POEM/LHM cohort remained unchanged, whereas the matched non-myotomy cohort continued to exhibit longer follow-up, with a mean of approximately 4.26 years and a median of 3.53 years ( Table 2 ).

**Table:** **Table 2** Follow-up duration before and after propensity score matching (years).

**Analysis stage**	**Cohort**	**Number of patients**	**Mean follow-up (years)**	**SD (years)**	**Median follow-up (years)**	**IQR (years)**
**Before PSM**	Myotomy	3,758	3.38	3.59	2.08	4.65
	Non-myotomy	14,428	3.90	3.59	3.08	5.15
**After PSM**	Myotomy	3,754	3.38	3.59	2.08	4.65
	Non-myotomy	3,754	4.26	3.61	3.53	5.21
IQR, interquartile range; PSM, propensity score matching; SD, standard deviation.						

### Clinical outcomes following myotomy compared with non-myotomy management

**Fig. 1:**
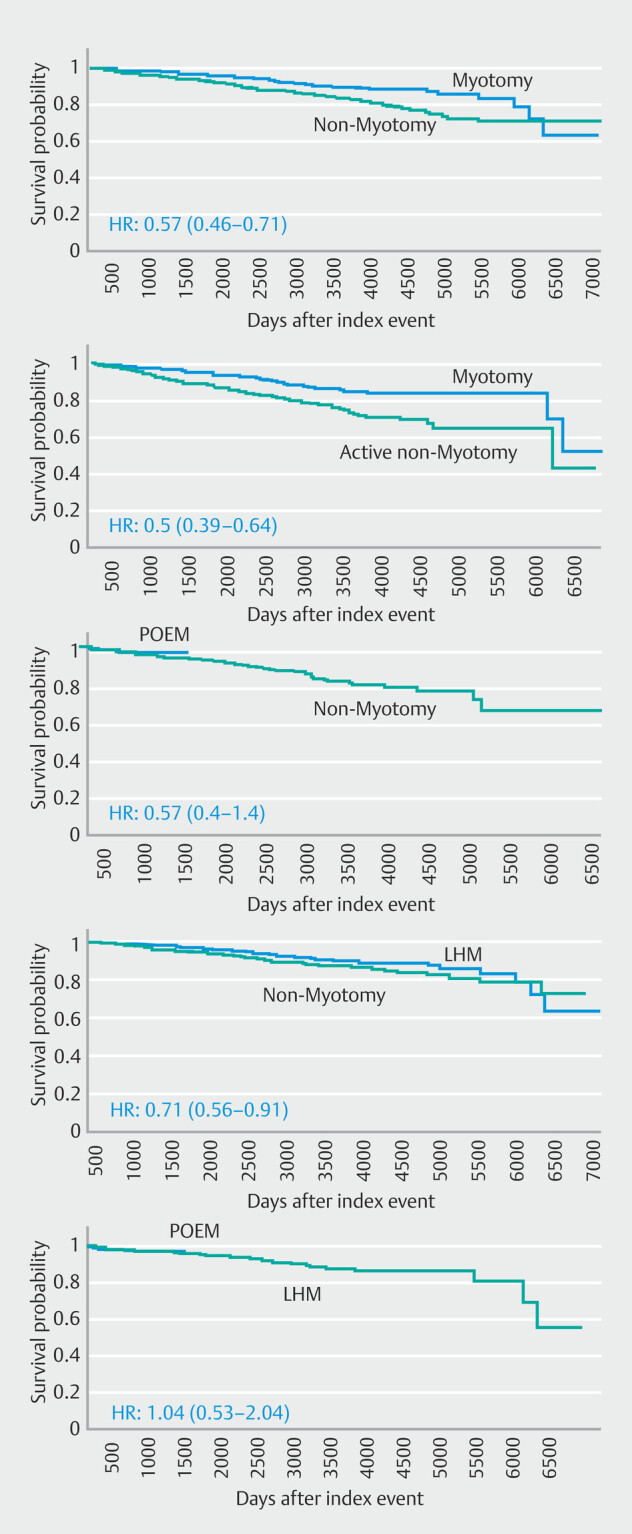
Kaplan-Meier curves comparing overall survival after propensity score matching for: 1) myotomy versus non-myotomy management; 2) myotomy versus active non-myotomy interventions; 3) peroral endoscopic myotomy (POEM) versus non-myotomy management; 4) laparoscopic Heller myotomy (LHM) versus non-myotomy management; and 5) POEM versus LHM. Hazard ratios (HRs) with 95% confidence intervals (CIs) are shown.

A total of 3,758 patients with achalasia underwent esophageal myotomy, whereas 14,428 were managed without myotomy, including medical management, endoscopic therapies, or observation. Following PSM, 3,754 patients remained in each cohort. During the follow-up period, EC occurred in 11 of 3,754 patients in the myotomy cohort, corresponding to an absolute risk of 0.29% ( Table 3 ). In comparison, 10 of 3,754 patients in the non-myotomy cohort developed EC, yielding an absolute risk of 0.27%. There was no statistically significant difference in EC incidence between the two groups (OR 1.1, 95% CI 0.47–2.6; *P* = 0.68). In contrast, patients who underwent myotomy had significantly lower risks of BE (2.13% vs 3.94%; OR 0.53, 95% CI 0.4–0.7; *P* < 0.001) and BE with dysplasia (0.27% vs 0.67%; OR 0.40, 95% CI 0.19–0.83; *P* = 0.01). Similarly, incidence of GERD was significantly lower in the myotomy cohort compared with the non-myotomy cohort (39.45% vs 50.88%; OR 0.63, 95% CI 0.58–0.69; *P* < 0.001) ( Table 3 ). All-cause mortality was also significantly reduced among patients undergoing myotomy (3.25% vs 7.22%; OR 0.43, 95% CI 0.35–0.54; *P* < 0.001). Kaplan-Meier curves demonstrated improved survival over time in the myotomy group ( Fig. 1 ).

**Table:** **Table 3** Clinical outcomes between myotomy and non-myotomy groups after propensity score matching.

**Outcome**	**Myotomy (POEM/LHM)** **cohort (n = 3,754)**	**Absolute risk**	**Non-myotomy cohort (n = 3,754)**	**Absolute risk**	**Odds ratio (95% CI)**	***P* value **
Esophageal cancer	11	0.29%	10	0.27%	1.1 (0.47–2.6)	0.683
Barrett’s esophagus	80	2.13%	148	3.94%	0.53 (0.4–0.7)	**< 0.001**
Barrett’s esophagus with dysplasia	10	0.27%	25	0.67%	0.4 (0.19–0.83)	**0.011**
GERD	1,481	39.45%	1,910	50.88%	0.63 (0.58–0.69)	**< 0.001**
All-cause mortality	122	3.25%	271	7.22%	0.43 (0.35–0.54)	**< 0.001**
CI, confidence interval; GERD, gastroesophageal reflux disease; LHM, laparoscopic Heller myotomy; POEM, peroral endoscopic myotomy.						

### Clinical outcomes following myotomy compared with active non-myotomy interventions

We compared patients who underwent esophageal myotomy (n = 3,758) with those who received active non-myotomy interventions (n = 2,257), including bougie dilation, endoscopic dilation, pneumatic dilation, and/or botulinum toxin injection. After PSM, each cohort comprised 1,894 patients. In the matched analysis, all-cause mortality was significantly lower in the myotomy cohort compared with the active non-myotomy group (4.54% vs 11.04%; OR 0.38, 95% CI 0.30–0.50; *P* < 0.001). Time-to-event analyses using Kaplan-Meier methods indicated improved overall survival among patients in the myotomy group ( Fig. 1 ). The incidence of BE did not differ significantly between the two groups (2.59% vs 3.06%; OR 0.84, 95% CI 0.57–1.24; *P* = 0.38). In contrast, myotomy was associated with a significantly lower risk of GERD compared with active non-myotomy management (43.30% vs 53.33%; OR 0.67, 95% CI 0.59–0.76; *P* < 0.001). EC events were rare in both groups, and comparative estimates could not be reliably reported due to small counts ( Table 4 ).

**Table:** **Table 4** Clinical outcomes after propensity score matching: Myotomy vs active non-myotomy.

**Outcome**	**Myotomy (POEM/LHM) cohort (n = 1,894)**	**Absolute risk**	Active non-myotomy cohort* (n = 1,894)	**Absolute risk**	**Odds ratio (95% CI)**	***P* value **
**All-cause mortality**	86	4.54%	209	11.04%	0.38 (0.30–0.50)	< 0.001
**Barrett’s esophagus**	49	2.59%	58	3.06%	0.84 (0.57–1.24)	0.377
**GERD**	820	43.30%	1,010	53.33%	0.67 (0.59–0.76)	< 0.001
*Active non-myotomy interventions included bougie dilation, endoscopic dilation, pneumatic dilation, and/or botulinum toxin injection. CI, confidence interval; GERD, gastroesophageal reflux disease; LHM, laparoscopic Heller myotomy; POEM, peroral endoscopic myotomy.						

### Risk of mortality, EC, and BE based on LES-directed therapy

EC events were rare in all subgroups, and detailed comparative estimates could not be reported due to small counts.

### Clinical outcomes: POEM vs non-myotomy

Before PSM, 1,159 patients who underwent POEM were compared with 14,428 patients managed without myotomy. After 1:1 PSM, 1,149 patients remained in each cohort. In the matched comparison, all-cause mortality was significantly lower in the POEM cohort compared with the non-myotomy cohort (1.22% vs 7.22%; OR 0.16, 95% CI 0.08–0.28; *P* < 0.001). POEM was also associated with a significantly lower incidence of BE (0.87% vs 4.27%; OR 0.19, 95% CI 0.11–0.39; *P* < 0.001). Similarly, risk of GERD was significantly reduced among patients treated with POEM compared with the non-myotomy cohort (38.38% vs 52.39%; OR 0.57, 95% CI 0.48–0.67; *P* < 0.001) ( Table 5 ).

**Table:** **Table 5** Clinical outcomes after propensity score matching: POEM vs non-myotomy.

**Outcome**	**POEM cohort (n = 1,149)**	**Absolute risk**	**Non-myotomy cohort (n = 1,149)**	**Absolute risk**	**Odds ratio (95% CI)**	***P* value **
All-cause mortality	14	1.22%	83	7.22%	0.16 (0.08–0.28)	**< 0.001**
Barrett’s esophagus	10	0.87%	49	4.27%	0.19 (0.11–0.39)	**< 0.001**
GERD	441	38.38%	602	52.39%	0.57 (0.48–0.67)	**< 0.001**
CI, confidence interval; GERD, gastroesophageal reflux disease; LHM, laparoscopic Heller myotomy; POEM, peroral endoscopic myotomy.						

### Clinical outcomes: Laparoscopic Heller myotomy versus non-myotomy

Before PSM, 2,634 patients who underwent LHM were compared with 14,428 patients managed without myotomy. After 1:1 PSM, 2,634 patients remained in each cohort. In the matched comparison, all-cause mortality was significantly lower in the LHM cohort compared with the non-myotomy cohort (4.10% vs 5.73%; OR 0.70, 95% CI 0.55–0.91; *P* = 0.006). Kaplan-Meier analysis showed superior survival in the LHM group over time ( Fig. 1 ). LHM was also associated with a modest but statistically significant reduction in incidence of BE (2.73% vs 3.91%; OR 0.69, 95% CI 0.51–0.94; *P* = 0.017). Similarly, risk of GERD was significantly lower among patients treated with LHM compared with the non-myotomy cohort (39.98% vs 52.47%; OR 0.60, 95% CI 0.54–0.67; *P* < 0.001) ( Table 6 ).

**Table:** **Table 6** Clinical outcomes after propensity score matching: LHM vs non-myotomy.

**Outcome**	**LHM cohort (n = 2,634)**	**Absolute risk**	**Non-myotomy cohort (n = 2,634)**	**Absolute risk**	**Odds ratio (95% CI)**	***P* value **
All-cause mortality	108	4.10%	151	5.73%	0.70 (0.55–0.91)	**0.006**
Barrett’s esophagus	72	2.73%	103	3.91%	0.69 (0.51–0.94)	0.017
GERD	1,053	39.98%	1,382	52.47%	0.60 (0.54–0.67)	**< 0.001**
CI, confidence interval; GERD, gastroesophageal reflux disease; LHM, laparoscopic Heller myotomy; POEM, peroral endoscopic myotomy.						

### Clinical outcomes: POEM vs LHM

Before PSM, 1,159 patients who underwent POEM were compared with 2,634 patients who underwent laparoscopic Heller myotomy. After 1:1 PSM, 1,101 patients remained in each cohort. In this exploratory matched analysis, all-cause mortality was significantly lower in the POEM cohort compared with the LHM cohort (1.27% vs 4.91%; OR 0.25, 95% CI 0.14–0.45; *P* < 0.001). Kaplan-Meier curves demonstrated a survival advantage for patients undergoing POEM ( Fig. 1 ). POEM was also associated with a significantly lower incidence of BE (0.91% vs 2.73%; OR 0.33, 95% CI 0.16–0.67; *P* = 0.001). In contrast, incidence of GERD did not differ significantly between patients treated with POEM and those treated with LHM (38.15% vs 41.78%; OR 0.86, 95% CI 0.73–1.02; *P* = 0.08) ( Table 7 ).

**Table:** **Table 7** Exploratory clinical outcomes after propensity score matching: POEM vs laparoscopic Heller myotomy.

**Outcome**	**POEM cohort (n = 1,101)**	**Absolute risk**	**LHM cohort (n = 1,101)**	**Absolute risk**	**Odds ratio (95% CI)**	***P* value **
All-cause mortality	14	1.27%	54	4.91%	0.25 (0.14–0.45)	**< 0.001**
Barrett’s esophagus	10	0.91%	30	2.73%	0.33 (0.16–0.67)	**0.001**
GERD	420	38.15%	460	41.78%	0.86 (0.73–1.02)	0.082
CI, confidence interval; GERD, gastroesophageal reflux disease; LHM, laparoscopic Heller myotomy; POEM, peroral endoscopic myotomy.						

### Predictors of esophageal cancer

**Fig. 2:**
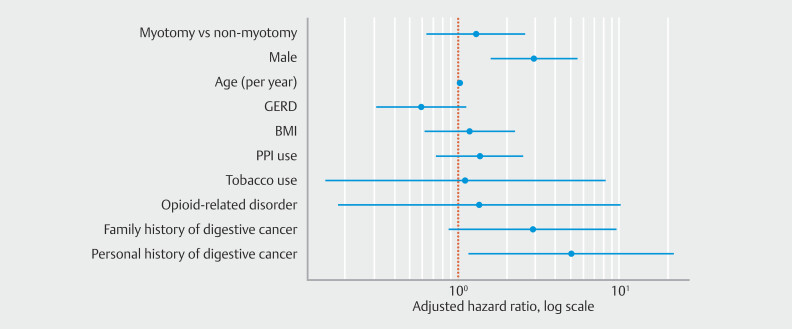
Cox regression model depicting hazard ratios (HRs) and 95% confidence intervals (CIs) for predictors of esophageal cancer in achalasia patients after myotomy.

Cox proportional hazards modeling identified male sex (aHR = 2.95; 95% CI 1.60–5.45; *P* < 0.001) and increasing age (aHR per year = 1.03; 95% CI 1.01–1.05; *P* = 0.009) as independent predictors of EC. A personal history of malignant neoplasm of the digestive organs was also significantly associated with an increased risk of EC (aHR = 5.05; 95% CI 1.19–21.51; *P* = 0.028). Family history of digestive organ malignancy showed a trend toward increased risk but did not reach statistical significance (aHR = 2.90; 95% CI 0.88–9.53; *P* = 0.08). In contrast, myotomy status, GERD, BMI, PPI use, tobacco use, opioid-related disorders, and alcohol use were not significantly associated with EC risk ( Fig. 2 ).

## Discussion

We investigated the association between esophageal myotomy (LHM or POEM) and risk of EC and mortality in patients with achalasia. In this large, real-world cohort, esophageal myotomy was associated with a similar short-term risk of EC but significantly lower all-cause mortality compared with non-myotomy management.

Several prior studies suggested a potential association between definitive achalasia therapy and lower EC incidence, particularly when compared with non-definitive treatments such as pneumatic dilation. A large case-control study of over 7,000 achalasia patients found that surgical myotomy as primary treatment was linked to a reduced risk of EC compared with pneumatic dilation 18 . Similarly, a retrospective study of more than 800 achalasia patients with a median follow-up of 72 months reported five new EC cases—all occurring in patients treated with pneumatic dilation; none were observed in those who underwent myotomy 19 . A Dutch cohort study of approximately 450 achalasia patients identified 15 EC cases after a mean follow-up of 11 years, with none having undergone esophageal myotomy 10 . A US veterans’ cohort similarly observed fewer EC cases among patients who underwent myotomy 20 . Conversely, one Italian cohort study reported that all 20 EC cases occurred in patients treated with myotomy 21 . However, since 80% of this cohort received myotomy, treatment selection bias may explain these findings rather than a causal relationship.

Collectively, prior studies have been limited by small event counts, heterogeneous follow-up durations, and nonrandom treatment allocation.

Importantly, in our matched analysis, we did not observe a statistically significant difference in EC incidence between myotomy and non-myotomy cohorts. This likely reflects the low absolute incidence of EC, limited event counts, and relatively short post-myotomy follow-up, rather than absence of a biological effect. Subgroup analyses by histologic subtype—including SCC and adenocarcinoma—were not feasible due to limitations in ICD-10 coding granularity and insufficient event numbers, precluding reliable classification of upper versus lower esophageal tumors. We hypothesize that myotomy may reduce EC risk by alleviating esophageal stasis and chronic mucosal inflammation, mechanisms thought to contribute predominantly to SCC. However, larger studies with longer follow-up and more granular histopathologic data are needed to clarify whether definitive achalasia therapy differentially affects EC subtypes.

Male sex, increasing age, and personal history of gastrointestinal cancers emerged as independent predictors of EC after myotomy in our cohort, consistent with prior research in both achalasia and general populations 9 10 16 21 22 23 24 25 26 . These shared risk factors highlight that EC risk in achalasia is influenced not only by treatment strategy but also by underlying patient characteristics, suggesting that individuals with these features may warrant closer surveillance regardless of whether they undergo myotomy.

In addition to cancer outcomes, we observed significantly lower rates of GERD and BE among patients undergoing esophageal myotomy compared with non-myotomy management. These findings should be interpreted with caution because GERD and achalasia share overlapping symptoms that may lead to diagnostic misclassification or miscoding in administrative data. This is particularly relevant given that esophageal myotomy—especially POEM—is known to be associated with higher rates of post-procedural GERD, reported in up to 40% to 60% of patients 27 28 29 30 , suggesting that the observed associations likely reflect limitations of coding rather than a true reduction in reflux-related disease.

Our study demonstrated that esophageal myotomy was associated with significantly lower all-cause mortality, a finding consistent across both POEM and LHM. Although data on mortality outcomes after myotomy are limited, both techniques are established, effective therapies that improve dysphagia and reduce achalasia-related complications 3 5 31 . The observed mortality benefit likely reflects factors beyond cancer prevention, including improved esophageal emptying, reduced aspiration risk, better nutritional status, and fewer hospitalizations related to food impaction or aspiration pneumonia. In addition, patients selected for definitive myotomy may have better baseline functional status and access to specialized care, which could independently contribute to improved survival. Accordingly, these findings demonstrate an association rather than a causal effect. In exploratory analyses, POEM was associated with lower mortality compared with LHM; however, this finding should be interpreted with caution. Temporal improvements in peri-procedural care, patient selection, endoscopists experience, and post-procedure follow-up may contribute to improved outcomes in more recent cohorts. Given the lack of granular data on disease severity and frailty, this comparison should be considered hypothesis-generating rather than evidence of procedural superiority. Overall, these results suggest that definitive treatment of achalasia with esophageal myotomy is associated with meaningful survival benefit, likely mediated through reductions in achalasia-related morbidity rather than direct effects on EC incidence

Our study has several notable strengths. First, it represents one of the largest cohorts to date evaluating the association between esophageal myotomy and EC risk in patients with achalasia, leveraging data from over 18,000 individuals across a large, multi-institutional, real-world dataset. This broad population enhances generalizability of our findings. Second, we used rigorous PSM to balance baseline characteristics, including age, sex, BMI, behavioral factors, comorbidities, and PPI use, thereby minimizing potential confounding and allowing for a more valid comparison between treatment groups. Third, we performed subgroup analysis to evaluate clinical outcomes between POEM and LHM separately compared with non-myotomy management. Fourth, we examined all-cause mortality in addition to cancer risk, offering a more comprehensive evaluation of the clinical impact of esophageal myotomy.

Our study has some limitations. Despite PSM, residual confounding and selection bias remain possible due to the retrospective design. In addition, use of deidentified database records limited access to detailed clinical variables, including achalasia subtype, disease duration, severity as well as procedure details such as POEM orientation (anterior versus posterior). Potential coding inaccuracies and underreporting of outcomes or comorbidities may exist. In addition, patients seeking care outside participating institutions may result in incomplete follow-up data. Patients undergoing myotomy may have been subject to more intensive surveillance, potentially increasing cancer detection. While both cohorts were balanced for the number of EGDs, residual differences in surveillance practices cannot be excluded. Follow-up duration was also shorter in the myotomy cohort compared with the non-myotomy group, which may affect detection of time-dependent outcomes such as EC. Although our time-to-event analyses account for varying follow-up durations, the overall follow-up period after myotomy remained relatively short, and development of EC may occur beyond the observed timeframe. Finally, the database lacks detailed quantification of tobacco and alcohol use, including amount and duration, which may influence risk assessment. Future prospective studies with longer follow-up and more granular clinical and histologic data are needed to better define treatment-specific and subtype-specific EC risk in patients with achalasia.

## Conclusions

In short-term follow-up, esophageal myotomy in patients with achalasia was associated with a similar incidence of EC but significantly lower all-cause mortality compared with non-myotomy management. These findings support the role of definitive achalasia therapy not only for symptom control but also for potential survival benefit. However, given the low absolute risk of EC and limitations of observational data, myotomy should not be considered a substitute for appropriate cancer surveillance in high-risk patients. Prospective studies with longer follow-up are needed to confirm these findings and to better define the impact of achalasia treatment on EC risk.

## Supplementary Material

Supplementary Material Zusatzmaterial
