# Supplementary material for: Esophageal myotomy and risk of esophageal cancer and mortality in achalasia: Real-world cohort study
Source: Endosc Int Open. 2026 Mar 16;14:a28014957. doi: 10.1055/a-2801-4957 (PMC13063302; doi:10.1055/a-2801-4957)
Supplement: Supplementary file 1 — Supplementary Material [file 10-1055-a-2801-4957_28148084.pdf]

**Supplementary Table 1** ICD-10-CM and CPT codes used to define diagnoses and procedures.

| Term | ICD-10, CPT, or RxNorm description | ICD-10, CPT, or RxNorm codes |
|---|---|---|
| Achalasia | Achalasia of cardia | ICD10CM: K22.0 |
| Motility study/manometry | Esophageal motility (manometric study of the esophagus and/or gastroesophageal junction) study with interpretation and report; with high resolution esophageal pressure topography | CPT: 0240T |
| | Esophageal motility (manometric study of the esophagus and/or gastroesophageal junction) study with interpretation and report | CPT: 1014295 |
| | esophageal motility (manometric) study with interpretation and report. | CPT: 91010 |
| | Esophageal motility (manometric study of the esophagus and/or gastroesophageal junction) study with interpretation and report | CPT: 1020556 |
| | Esophageal motility | CPT: 78258 |
| Myotomy | Lower esophageal myotomy, transoral (ie, peroral endoscopic myotomy (POEM) | CPT: 43497 |
| | Laparoscopy, surgical, esophagomyotomy (Heller type), with fundoplasty, when performed | CPT: 43279 |
| | Esophagomyotomy (Heller type); abdominal approach | CPT: 43330 |
| | Esophagomyotomy (Heller type) | CPT: 1007313 |
| | Esophagomyotomy (Heller type); thoracic approach | CPT: 43331 |
| | Thoracoscopy, surgical; with esophagomyotomy (Heller type) | CPT: 32665 |
| Esophageal cancer | Malignant neoplasm of esophagus | ICD10CM: C15 |
| | Malignant neoplasm of lower third of esophagus | ICD10CM: C15.5 |
| | Malignant neoplasm of middle third of esophagus | ICD10CM: C15.4 |
| | Malignant neoplasm of upper third of esophagus | ICD10CM: C15.3 |
| | Malignant neoplasm of overlapping sites of esophagus | ICD10CM: C15.8 |

| | | |
|---|---|---|
| Esophagectomy | Esophagectomy | SNOMED: 45900003 |
| | Esophagectomy, distal two-thirds, laparoscopic mobilization with proximal gastrectomy (Ivor Lewis esophagectomy) | CPT: 43287 |
| | Esophagectomy, total/near total, thoracoscopic mobilization with laparoscopic proximal gastrectomy (McKeown esophagectomy) | CPT: 43288 |
| | Esophagectomy, total/near total, laparoscopic mobilization with proximal gastrectomy (transhiatal esophagectomy) | CPT: 43286 |
| | Total or near total esophagectomy, with thoracotomy; with pharyngogastrostomy or cervical esophagogastrostomy, with or without pyloroplasty (ie, McKeown esophagectomy or tri-incisional esophagectomy) | CPT: 43112 |
| | Partial esophagectomy, distal two-thirds, with thoracotomy and separate abdominal incision, with or without proximal gastrectomy | CPT: 1007230 |
| | Total or near total esophagectomy, without thoracotomy | CPT: 1007223 |
| | Total or near total esophagectomy, without thoracotomy; with pharyngogastrostomy or cervical esophagogastrostomy, with or without pyloroplasty (transhiatal) | CPT: 43107 |
| | Total or near total esophagectomy, with thoracotomy | CPT: 1007226 |
| | Partial esophagectomy, thoracoabdominal or abdominal approach, with or without proximal gastrectomy | CPT: 1007234 |
| | Total or partial esophagectomy, without reconstruction (any approach), with cervical esophagostomy | CPT: 43124 |
| | Total or near total esophagectomy, without thoracotomy; with colon interposition or small intestine reconstruction, including intestine mobilization, preparation and anastomosis(es) | CPT: 43108 |
| | Partial esophagectomy, cervical, with free intestinal graft, including microvascular anastomosis, obtaining the graft and intestinal reconstruction | CPT: 43116 |
| Barrett's esophagus | Barrett's esophagus | ICD10CM: K22.7 |
| | Barrett's esophagus without dysplasia | ICD10CM: K22.70 |
| | Barrett's esophagus with dysplasia | ICD10CM: K22.71 |

| | | |
|---|---|---|
| | Barrett's esophagus with dysplasia, unspecified | ICD10CM: K22.719 |
| | Barrett's esophagus with low-grade dysplasia | ICD10CM: K22.710 |
| | Barrett's esophagus with high-grade dysplasia | ICD10CM: K22.711 |
| Other cancers | Malignant neoplasms of head, face and neck | ICD10CM: C76.0 |
| | Malignant neoplasms of breast | ICD10CM:C50 |
| | Malignant neoplasms of bronchus and lung | ICD10CM:C34 |
| | Malignant neoplasms of stomach | ICD10CM: C16 |
| Other non-myotomy interventions | Dilation of esophagus with intraluminal device, via natural or artificial opening | ICD-10-PCS: 0D757DZ |
| | Dilation of esophagogastric junction, via natural or artificial opening | ICD-10-PCS: 0D748ZZ |
| | Dilation of lower esophagus, via natural or artificial opening endoscopic | ICD-10-PCS: 0D738ZZ |
| | Dilation of esophagogastric junction with intraluminal device, via natural or artificial opening | ICD-10-PCS: 0D737ZZ |
| | Dilation of lower esophagus, via natural or artificial opening | ICD-10-PCS: 0D737DZ |
| | Dilation of lower esophagus with intraluminal device, via natural or artificial opening | ICD-10-PCS: 0D738DZ |
| | Dilation of esophagus; by unguided sound or bougie, single or multiple passes. | CPT: 43450 |
| | Esophagoscopy, flexible, transoral; with directed submucosal injection(s), any substance. | CPT: 43201 |
| | Esophagogastroduodenoscopy, flexible, transoral; with directed submucosal injection(s), any substance | CPT: 43236 |
| GERD | Gastroesophageal reflux disease | ICD-10: K21 |
| Cirrhosis | Hepatic fibrosis | ICD-10: K74.0 |

| | | |
|---|---|---|
| Chronic kidney disease | Chronic kidney disease (CKD) | ICD-10: N18 |
| COPD | Chronic obstructive pulmonary disease | ICD-10: J44 |
| Opioid use | Opioid related disorders | ICD-10: F11 |
| Heart failure | Heart failure | ICD-10: I50 |
| Diabetes mellitus | Type 2 diabetes mellitus | ICD-10: E11 |
| Hypertension | Hypertensive diseases | ICD-10: I10-I1A |
| Hyperlipidemia | Hyperlipidemia, unspecified | ICD-10: E78.5 |
| Proton pump inhibitors | Proton pump inhibitors | RxNorm: A02BC |
| Body mass index | BMI | TNX curated: 9038 |
| Tobacco use | Tobacco use | ICD-10-CM: Z72.0 |
| alcohol use | Alcohol use, unspecified | ICD-10-CM: F10.9 |
| Opioid related disorders | Opioid related disorders | ICD-10-CM: F11 |
| Family history of gastrointestinal cancer | Family history of malignant neoplasm of digestive organs. | ICD-10-CM: Z80.0 |
| Personal history of gastrointestinal cancer | Personal history of malignant neoplasm of digestive organs | ICD-10-CM: Z85.0 |
| Diaphragmatic hernia | Diaphragmatic hernia | ICD-10-CM: K44 |
| EGD | Esophagogastroduodenoscopy, flexible, transoral CPT | CPT: 1007260 |
